# Delta Neutrophil Index in Suspected Septic Arthritis: A Diagnostic Accuracy Study

**DOI:** 10.3390/jcm15020840

**Published:** 2026-01-20

**Authors:** Hüseyin Emre Tepedelenlioğlu, Hilmi Alkan, Tural Talıblı, Ünal Erkanov Hüseyinov, Ferid Abdulaliyev, Erkan Akgün, Vedat Biçici

**Affiliations:** 1 Department of Orthopedics and Traumatology, Ankara Etlik City Hospital, Ankara 06170, Türkiye; hilmi_alkan@hotmail.com (H.A.); turaltalibli50@gmail.com (T.T.); yunal776@gmail.com (Ü.E.H.); drorthopedic.akgun@gmail.com (E.A.); drvedatbicici@gmail.com (V.B.); 2 Department of Orthopedics and Traumatology, Sorgun State Hospital, Yozgat 66700, Türkiye; feridabduleliyev@gmail.com

**Keywords:** septic arthritis, delta neutrophil index, diagnostic accuracy, ROC analysis

## Abstract

**Background/Objectives:** Septic arthritis of native joints is an orthopedic emergency in which rapid discrimination from non-infectious arthritis is crucial. Because cartilage damage can occur within hours, urgent irrigation and debridement are often pursued on an emergency basis (ideally within the first 6–8 h) of presentation, underscoring the need for rapidly available biomarkers. The delta neutrophil index (DNI) quantifies circulating immature granulocytes and may complement conventional inflammatory biomarkers such as C-reactive protein (CRP), erythrocyte sedimentation rate (ESR), white blood cell count (WBC), and procalcitonin (PCT). We evaluated the diagnostic performance of DNI for native-joint septic arthritis against both microbiologic and clinical reference standards. **Methods:** We retrospectively analyzed 85 adults who underwent surgical irrigation and debridement for suspected native joint septic arthritis at a tertiary center. Serum CRP, ESR, WBC, DNI, and PCT (available in 67 patients) were recorded together with synovial leukocyte counts. Infection status was defined using either positive synovial culture (microbiologic reference) or clinical adjudication according to the Guideline for management of septic arthritis in native joints (SANJO). Diagnostic performance was assessed using receiver operating characteristic (ROC) curves and the area under the ROC curve (AUC); exploratory cut-offs were identified by the Youden index, and pairwise AUCs were compared using DeLong’s test. **Results:** Synovial leukocyte analysis was highly sensitive but poorly specific (sensitivity 92.9%, specificity 10.3%). Against culture, DNI showed the highest discrimination (AUC = 0.914), exceeding CRP (0.687), ESR (0.643), WBC (0.648), and PCT (0.697); DeLong ΔAUC vs. CRP 0.227 (*p* < 0.001), ESR 0.270 (*p* < 0.001), WBC 0.266 (*p* < 0.001), PCT 0.227 (*p* = 0.001). At pre-specified cut-offs, DNI showed the most balanced sensitivity/specificity (94.3%/84.0%), corresponding to a negative predictive value (NPV) of 95.5% (42/44) and a positive predictive value (PPV) of 80.5% (33/41) against culture in this cohort. Against clinical infection, DNI outperformed others (AUC:0.921; ΔAUC vs. CRP = 0.204, ESR = 0.343, WBC = 0.244, PCT = 0.295; all *p* < 0.001). As a rule-in threshold, DNI ≥ 0.6 yielded a specificity of 100% with a sensitivity of 73.2%. In culture-negative patients (infected n = 21, uninfected n = 29), DNI remained discriminatory (AUC 0.80, *p* < 0.001), whereas other biomarkers were not. **Conclusions:** DNI demonstrated superior diagnostic accuracy compared with conventional inflammatory biomarkers. As a rapid parameter available with the initial complete blood count, DNI may support early risk stratification and rule-in decisions within the first hours of presentation; however, it should be used as a supplementary indicator alongside synovial fluid analysis and clinical assessment rather than as a stand-alone diagnostic tool.

## 1. Introduction

Septic arthritis is a time-critical orthopedic emergency [1]. Because proteolytic enzymes and the host inflammatory response can cause irreversible cartilage damage within hours, prompt evaluation and expedited joint irrigation and debridement are commonly pursued on an emergency basis, ideally within the first 6–8 h of presentation [2]. It occurs due to the hematogenous spread or direct inoculation of pathogenic microorganisms into the joint, leading to significant morbidity and complications. Septic arthritis is one of the few conditions in orthopedics that requires immediate surgical intervention. However, it can often be confused with non-infectious diseases such as rheumatologic conditions, inflammatory arthritis, and crystal arthropathy, which can increase comorbidity due to unforeseen unnecessary surgeries.

Distinguishing septic arthritis from other inflammatory arthritis is essential to prevent unnecessary surgery while also ensuring that an orthopedic emergency is not overlooked. Currently, the diagnosis of septic arthritis is made through comprehensive medical history, physical examination findings, blood tests, and joint aspiration samples [3]. However, synovial fluid cultures typically require 48–72 h, meaning that critical surgical decisions are often made before definitive microbiology is available; this creates a clinical need for rapidly available biomarkers that can support early decision-making. Still, there are limitations in the clinical and laboratory diagnostic values that are routinely used in diagnosis. The fundamental principle of treating bacterial septic arthritis is prompt joint irrigation and debridement, followed by targeted antibiotic therapy.

A ‘left shift’ in peripheral blood has long been associated with bacterial infection. The Delta Neutrophil Index (DNI) is an automated surrogate of this response reported by certain hematology analyzers. On ADVIA-type platforms, DNI is derived from the difference between measurements obtained in the myeloperoxidase (MPO) channel and the nuclear lobularity channel, thereby estimating the fraction of circulating immature granulocytes (IG; promyelocytes, myelocytes, metamyelocytes, and band forms) [4]. Because DNI reflects early myeloid activation, it may rise earlier than hepatic acute-phase reactants such as CRP and ESR and may be less confounded by chronic inflammatory conditions. DNI has been evaluated in systemic infections and in musculoskeletal infections (e.g., foot and ankle infections), and some studies have reported stronger discrimination than conventional markers such as WBC, ANC, ESR, and CRP [5,6,7,8]. Nevertheless, DNI’s diagnostic utility in native-joint septic arthritis remains uncertain.

The aim of this study was to compare the diagnostic performance of the DNI with routine inflammatory biomarkers, including CRP, ESR, WBC, and PCT, for native joint septic arthritis, using both microbiological culture and the Guideline for management of septic arthritis in native joints (SANJO)-based clinical adjudication as reference standards.

## 2. Materials and Methods

### 2.1. Study Design and Setting

This retrospective diagnostic accuracy study was conducted at Ankara Etlik City Hospital, a tertiary referral center, in accordance with the Declaration of Helsinki. The protocol was approved by the institutional ethics committee (No: AEŞH-BADEK-2024-319, date: 25 September 2024). Because inclusion required surgical irrigation and debridement for suspected septic arthritis, the cohort represents an enriched population with a high pre-test probability of infection.

### 2.2. Participants

Between November 2022 and September 2024, 100 adult (>18 y) patients presenting with a preliminary diagnosis of acute septic arthritis and undergoing surgery at a single tertiary care center (Ankara Etlik City Hospital) were examined.

**Figure 1:**
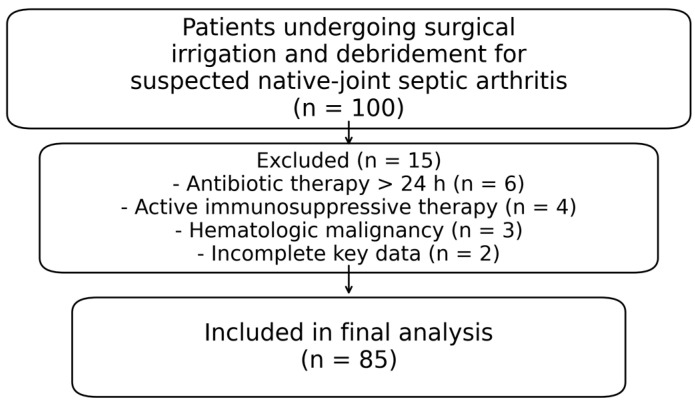
Patient enrollment and study flow.

Exclusion criteria included prior antibiotic therapy exceeding 24 h (n = 6), active immunosuppressive therapy (n = 4), hematologic malignancy (n = 3), and incomplete key laboratory or outcome data (n = 2). Exclusion criteria were applied hierarchically and were mutually exclusive. In total, 15 patients were excluded (13 meeting clinical exclusion criteria and 2 due to incomplete data), leaving 85 patients for analysis (Figure 1).

### 2.3. Biomarker Measurement

Venous blood samples were obtained as part of the routine preoperative workup within 2 h prior to surgery; when multiple measurements were available, the last value prior to incision was used. The timing of symptom onset relative to sampling was abstracted from the medical record when documented; however, symptom duration was inconsistently recorded and was not used in primary analyses. Synovial fluid was aspirated as early as possible after presentation and before surgical irrigation and debridement; aspirations were performed in the emergency department before transfer to the operating room. Synovial leukocyte counts were categorized using SANJO-recommended thresholds (<50,000; >50,000 cells/µL) [9]. Patients were categorized as infected or uninfected according to SANJO-based clinical criteria and microbiologic culture. Serum biomarkers recorded were WBC, CRP, PCT, ESR, and DNI. Routine laboratory reference cut-off values for WBC, CRP, PCT, and ESR were 4.5–11 × 10^9^/L, 5 mg/L, 0.5 ng/mL, and 14 mm/h, respectively. DNI was measured using an automated counter (ADVIA 120; Siemens Healthcare Diagnostics, Bayer, Whippany, NJ, USA). For transparency, DNI on this platform is reported as a percentage and is calculated from two independent analyzer channels: a peroxidase-based MPO channel that captures MPO-reactive granulocyte populations (including immature forms) and a nuclear lobularity channel that classifies mature polymorphonuclear neutrophils; the difference between these channel-derived fractions provides an estimate of circulating immature granulocytes [4]. For prespecified rule-in analyses, we used the manufacturer-provided positivity threshold of 0.6% [10]. This lower threshold is intended to flag early myeloid left shift and may be lower in localized, early infections than thresholds (~5–6%) reported in cohorts with bacteremia or severe systemic infection; therefore, the diagnostic cut-off should be considered platform- and context-specific and requires external validation. Because PCT was not routinely requested, analyses involving PCT were treated as secondary and were restricted to the 67 patients for whom this measurement was available (complete-case analysis). We did not impute missing PCT values, and the potential for selection bias related to clinician-driven ordering was acknowledged in the Section 4.

### 2.4. Reference Standards and Clinical Adjudication

Microbiologic reference standard: Synovial fluid was cultured using routine aerobic/anaerobic methods. A positive synovial fluid culture from a preoperative aspiration or an intraoperative specimen obtained prior to irrigation was considered microbiologically confirmed septic arthritis. Clinical reference standard (SANJO-based): In addition to culture, infection status was adjudicated retrospectively using SANJO diagnostic recommendations, incorporating (i) macroscopic purulence (aspirate or intraoperative findings), (ii) synovial leukocyte count and polymorphonuclear (PMN) predominance, (iii) Gram stain findings, (iv) imaging and operative findings when available, and (v) documented clinician decision to treat as septic arthritis (e.g., completion of an antibiotic course) versus an alternative non-infectious diagnosis. Adjudication was performed independently by two orthopedic surgeons using a standardized abstraction form; serum biomarker values under evaluation (DNI, CRP, ESR, WBC, and PCT) were masked during adjudication to minimize incorporation bias. Disagreements were resolved by consensus. Because synovial leukocyte counts are part of SANJO recommendations, analyses involving synovial leukocytes should be interpreted with awareness of potential incorporation effects.

### 2.5. Sample-Size Considerations

We did not perform an a priori sample size calculation because the study was retrospective and designed as an exploratory diagnostic accuracy analysis. We therefore report 95% confidence intervals for AUC estimates and interpret cut-off findings as hypothesis-generating.

### 2.6. Statistical Analysis

The statistical analysis of the study data was conducted at a 95% confidence level, and results with a *p*-value < 0.05 were considered statistically significant. Normality was assessed using the Shapiro–Wilk test; as biomarkers were non-normally distributed, Mann–Whitney U tests were applied for two-group comparisons (culture positive vs. negative) and Kruskal–Wallis tests for multi-group scenarios when required. Associations between categorical variables (e.g., sex, affected side) were examined with the Chi-square test. Diagnostic performances of DNI, CRP, ESR, and WBC were compared using receiver operating characteristic (ROC) curve analysis (higher values assumed positive). Area Under the Curve (AUC) values quantified discrimination, and pairwise AUC differences were evaluated using DeLong’s test; AUC confidence intervals were estimated using bootstrap resampling. Exploratory optimal cut-offs were identified using the Youden index; these cut-offs were not internally validated (e.g., bootstrapping/cross-validation), and corresponding sensitivity/specificity estimates may be optimistically biased. To assess potential selection bias related to missing PCT values, baseline characteristics were compared between patients with available and missing PCT measurements using Fisher’s exact test for categorical variables, Welch’s *t*-test for age, and Mann–Whitney U tests for laboratory parameters. In addition, multivariable logistic regression was performed to evaluate whether the DNI was independently associated with culture positivity after adjustment for age, sex, diabetes mellitus, CRP, and WBC. The statistical and logistic regression analysis of the data was performed using SPSS version 22.0.

## 3. Results

The demographic characteristics and outcomes of patients were summarized in Table 1. The distributions of age, sex, and side between the culture-positive group and the negative group were similar both between the culture-positive/negative groups and between the clinically infected/uninfected groups (*p* > 0.05). On the other hand, while CRP, ESR, and DNI levels were significantly higher in culture-positive cases (*p* < 0.05), WBC and PCT levels showed no significant difference (*p* > 0.05). When comparing synovial fluid results, a high synovial leukocyte count was observed in both culture-negative cases (94%) and culture-positive cases (89%), with no significant difference in distribution according to culture status (*p* = 0.439). Similar findings were obtained based on SANJO criteria for infected versus uninfected status (*p* = 0.686). The sensitivity of synovial leukocyte results was 92.9%, and the specificity was 10.3%.

Baseline characteristics stratified by PCT availability are shown in Table 2. Compared with patients without PCT measurements (n = 18), those with PCT measurements (n = 67) had a higher prevalence of diabetes mellitus (37.3% vs. 5.6%, *p* = 0.009) and hypertension (41.8% vs. 11.1%, *p* = 0.024), whereas age, sex, joint distribution, culture positivity, and inflammatory markers were similar.

Among culture-negative patients with an adjudicated clinical diagnosis (infected n = 21; uninfected n = 29), only DNI values were significantly higher in infected cases (*p* < 0.001) and demonstrated the best performance in predicting clinical infection. No significant differences were observed in CRP, ESR, WBC, and PCT levels (*p* = 0.061, *p* = 0.827, *p* = 0.227, and *p* = 0.769, respectively). In ROC analyses using clinical infection as the outcome, DNI achieved the highest overall accuracy (AUC = 0.80). CRP showed moderate performance (AUC = 0.66). ESR performed poorly (AUC = 0.47), whereas WBC favored sensitivity over specificity (AUC = 0.64). PCT measurement did not show a difference between infected and non-infected individuals in the current subgroup and was not discriminatory (AUC = 0.50) (Table 3).

**Figure 2:**
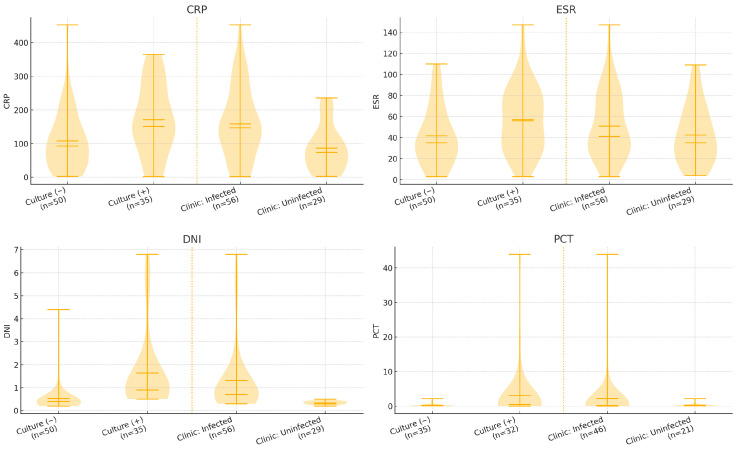
Violin plots for biomarkers based on culture and clinical results.

When biomarkers were examined at pre-specified cut-off values, DNI demonstrated the most balanced performance against culture (sensitivity 94.3%, specificity 84.0%). This yielded an NPV of 95.5% (42/44), supporting a rule-out interpretation when DNI is below the threshold in this high-pretest-probability surgical cohort. PCT (≥0.5 ng/mL) showed a rule-in pattern with high specificity (91.4%) but low sensitivity (46.9%). When SANJO-based clinical infection status was used as the reference standard, DNI (≥0.6) yielded a specificity 100% with a sensitivity 73.2%. In contrast, CRP and ESR were highly sensitive (98.2% and 89.3%, respectively) but poorly specific (10.3% and 13.8%, respectively). WBC showed modest sensitivity and specificity (60.7% and 65.5%). PCT maintained high specificity (95.2%) but low sensitivity (37.0%). Full results were provided in Table 4; biomarker distributions by culture and clinical status were shown in Figure 2.

**Figure 3:**
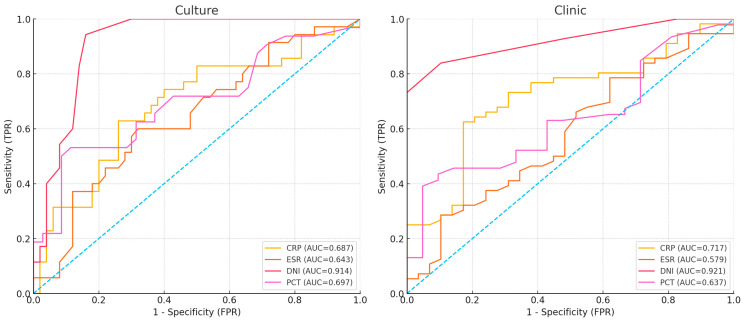
ROC curve analysis.

In the ROC curve analysis based on culture results, the DNI showed an AUC of 0.914, significantly outperforming other markers. According to the DeLong test, the AUC of DNI was higher than that of CRP by ΔAUC = 0.227 (*p* < 0.001), ESR by ΔAUC = 0.271 (*p* < 0.001), WBC by ΔAUC = 0.266 (*p* < 0.001), and PCT by ΔAUC = 0.217 (*p* = 0.001) (Table 5). According to the clinical presentation, DNI demonstrated the best performance with an AUC of 0.921. The DeLong test revealed that the AUC of DNI was higher than CRP by ΔAUC = 0.204 (*p* < 0.001); ESR by ΔAUC = 0.342 (*p* < 0.001); WBC by ΔAUC = 0.244 (*p* < 0.001); and PCT by ΔAUC = 0.284 (*p* < 0.001) (Table 6) (Figure 3).

In multivariable logistic regression using culture positivity as the dependent variable, DNI remained independently associated with culture positivity (adjusted odds ratio [aOR] per 0.1 increase: 1.21; 95% CI: 1.06–1.38; *p* = 0.006) after adjustment for age, sex, diabetes mellitus, CRP, and WBC. In a sensitivity analysis restricted to patients with available PCT measurements (n = 67) and additionally adjusting for log(1 + PCT), DNI remained significant (aOR per 0.1 increase: 1.22; 95% CI: 1.05–1.43; *p* = 0.011).

## 4. Discussion

Septic arthritis is most commonly observed in the knee and hip joints, although it can also occur less frequently in the hand, elbow, shoulder, foot, and ankle. In the differential diagnosis of acute septic arthritis, an acute rheumatoid flare should particularly be considered. In our study, we examined the parameters of patients who presented with a preliminary diagnosis of septic arthritis. The study excluded patients who presented with a diagnosis of acute rheumatoid flare, and the sensitivity and specificity of the DNI were compared with other infection parameters. In a study conducted by Pyo et al. [11], the DNI values were compared between patients presenting with septic arthritis and acute gout attack, and it was found that DNI was the most powerful independent predictor for septic arthritis. Our study also found that DNI had a stronger predictive value compared to other parameters.

The most frequently isolated pathogen in our culture-positive cases was *S. aureus*, consistent with prior reports [12]. Culture-negative septic arthritis is well recognized and may reflect prior antibiotic exposure, low bacterial burden, or fastidious organisms, among other factors. In our cohort, 58.8% of patients were culture negative, underscoring the practical need for adjunctive tools that can support early decision-making when microbiology is non-diagnostic [13,14]. In this context, DNI remained discriminatory in culture-negative patients who were clinically adjudicated as infected, whereas conventional biomarkers did not.

One of the key parameters used in the diagnosis of septic arthritis is the amount of leukocyte count in the synovial fluid. According to SANJO, a synovial white blood cell count > 50,000 cells/µL is suggestive of septic arthritis but is not sufficient alone for diagnosis [8], whereas a low synovial white blood cell count decreases the post-test probability but cannot exclude infection [15]. Consistent with this guidance, synovial leukocyte counts in our enriched surgical cohort showed high sensitivity but limited specificity, supporting the need to interpret synovial cell counts together with microbiology and the overall clinical context [16]. The very low specificity observed in our study likely reflects spectrum effects in an enriched surgical cohort, in which severe inflammatory mimics (crystal arthropathy, reactive synovitis, rheumatoid flare, and post-traumatic effusions) can present with markedly elevated synovial leukocyte counts. Accordingly, synovial WBC should be interpreted as a supportive, non-definitive marker, particularly early in the disease course and in the presence of inflammatory comorbidity.

The microbiologic reference standard for septic arthritis remains synovial fluid culture, but culture results may be negative despite true infection and typically require time for processing. Because emergent surgical debridement may be required before definitive culture results are available, clinicians often integrate macroscopic purulence, synovial leukocyte count/differential, Gram stain, and the clinical course when making treatment decisions [17]. In our cohort, culture-negative presentations included non-infectious etiologies (e.g., inflammatory flares or reactive synovitis), highlighting the limited specificity of synovial leukocyte counts in isolation. DNI may therefore serve as an adjunct biomarker to support early decision-making, but it should not replace synovial analysis or microbiologic testing [12].

Differentiating septic arthritis from inflammatory arthritis is of major clinical importance. The laboratory parameters commonly used for this purpose are CRP, ESR, and WBC. However, none of these markers have specific cut-off values for diagnostic use with high specificity. In our exploratory ROC-based cut-off analyses (Youden index), the estimated thresholds were 148.52 for CRP, 57 for ESR, and 10.35 for WBC. In the same exploratory framework, DNI showed a higher AUC than the other biomarkers. Because these cut-offs and performance estimates were derived and evaluated within the same sample without internal validation, they should be interpreted as hypothesis-generating and require external validation before clinical adoption.

DNI primarily reflects the fraction of immature granulocytes. Elevated DNI has been associated with infection severity and mortality [18,19]. Moon et al. [9] evaluated DNI in pneumonia-related sepsis and reported prognostic utility for 28-day mortality. Similarly, Pyo et al. [20] reported a DNI value of 6.4 in a patient with septic arthritis and bacteremia, and other studies in bacteremia/sepsis cohorts have reported thresholds in a similar range (~5.2–6.5) [16,21]. In our cohort, the highest DNI value observed was 5.9, and there were no clinical signs of bacteremia. Importantly, these higher thresholds were derived in populations with systemic infection and are intended to capture marked myeloid left shift. By contrast, the present study focused on early decision-making in suspected localized native-joint infection, and we therefore used the manufacturer-provided ADVIA threshold of 0.6% as a prespecified cut-off; this threshold also matched the Youden-derived cut-off in our data. Nonetheless, DNI thresholds are analyzer- and context-dependent and require external validation.

When examining the role of PCT in the diagnosis of septic arthritis, a retrospective study conducted by West et al. on 53 patients reported cut-off values for PCT as 0.25 and 0.32, respectively, concluding that PCT was superior to WBC, ESR, and CRP in terms of specificity and sensitivity [22]. Similarly, Zhao et al. concluded that PCT is more valuable than CRP for the diagnosis of septic arthritis [23]. PCT performed less well than expected in our cohort, although its specificity was found to be higher than that of CRP and WBC. This may partly reflect selection bias, as PCT was not measured in all patients and was more likely to be ordered in those with systemic signs of sepsis. Moreover, the standard cut-off of 0.5 ng/mL was originally derived for systemic bacterial infection and may be too high for isolated native joint infection, resulting in low sensitivity in our study.

Several limitations of this study should be acknowledged. First, the retrospective, single-center design may introduce selection and information bias. Second, because inclusion required surgical irrigation and debridement, the cohort is enriched with a high pretest probability of septic arthritis; this spectrum bias may inflate diagnostic performance and limit generalizability to broader emergency department populations, including patients managed non-operatively. Third, symptom duration relative to biomarker sampling was not consistently documented in the medical record, precluding time-from-onset analyses. Fourth, while we masked serum biomarker values during SANJO-based adjudication, synovial leukocyte counts are part of SANJO diagnostic recommendations and may introduce incorporation effects for analyses that evaluate synovial leukocytes. Fifth, PCT was available in only 67/85 patients because testing was clinician-driven; this missingness may introduce selection bias, and we therefore interpret PCT findings cautiously. Sixth, exploratory cut-offs were optimized on the same sample without internal validation; therefore, sensitivity/specificity estimates may be optimistically biased and require external validation. Finally, DNI reporting is dependent on analyzer-specific hematology platforms (e.g., Siemens ADVIA), which may limit adoption in centers without compatible equipment; in such settings, immature granulocyte parameters reported by other analyzers may be considered but were not evaluated in this study. Despite these limitations, DNI is rapidly available when supported by the local hematology platform and may provide incremental diagnostic value as an adjunct to synovial evaluation.

## 5. Conclusions

This study demonstrated that DNI achieved the highest discriminatory performance for septic arthritis among the evaluated biomarkers. In this surgical cohort, a DNI value ≥0.6% was highly specific and supported early risk stratification when interpreted alongside synovial fluid analysis and clinical assessment. Because the cohort was enriched and cut-offs were derived in an exploratory framework, these findings should be considered hypothesis-generating and warrant validation in broader emergency department populations.

## Figures and Tables

**Table 1:** Demographics of patients.

Variable	Value	*p* Value
Age (mean ± SD)	56.87 ± 24.73	0.054
Sex		
Male	47 (57.3%)	0.621
Female	38 (42.7%)	
Side		
Right	44 (51.8%)	0.991
Left	39 (45.9%)	
Bilateral	2 (2.4%)	
Culture: Negative	50 (58.8%)	
Infected	21 (42.0%)	
Uninfected	29 (58.0%)	
Culture: Positive	35 (41.2%)	
Pathogen: *S. aureus*	24 (68.5%)	
Pathogen: *S. epidermidis*	3 (8.7%)	
Pathogen: *S. dysgalactia*	1 (2.8%)	
Pathogen: *E. coli*	7 (20.0%)	
Joint		
Knee	69 (81.1%)	
Elbow	4 (4.6%)	
Shoulder	5 (5.9%)	
Hip	3 (3.6%)	
Wrist	1 (1.2%)	
Ankle	3 (3.6%)	
Biomarkers		
CRP (mg/L)	134.26 ± 100.79	0.005
ESR (mm/h)	48.02 ± 30.70	0.025
WBC (×10^3^/µL)	12.63 ± 5.07	0.226
DNI	0.99 ± 1.29	<0.001
PCT (ng/mL)	1.60 ± 5.95	0.069

**Table 2:** Baseline characteristics stratified by PCT availability.

Variable	PCT Available (n = 67)	PCT Missing (n = 18)	*p* Value
Age (years)	58.54 ± 24.69	51.67 ± 25.42	0.315
CRP (mg/L)	121.56 [67.37–188.87]	97.19 [23.49–198.49]	0.498
ESR (mm/h)	42.00 [27.50–71.50]	33.50 [16.00–59.25]	0.289
WBC (×10^3^/µL)	10.94 [9.77–13.02]	12.47 [10.20–17.24]	0.111
DNI	0.50 [0.40–0.85]	0.60 [0.33–1.05]	0.713
Male sex	37 (55.2%)	10 (55.6%)	1.000
Knee joint	55 (82.1%)	14 (77.8%)	0.737
Diabetes mellitus	25 (37.3%)	1 (5.6%)	0.009
Hypertension	28 (41.8%)	2 (11.1%)	0.024
Culture positive	24 (35.8%)	7 (38.9%)	0.791

**Table 3:** ROC performance of biomarkers in distinguishing clinical infection in culture-negative patients.

Biomarker	n	AUC (95% CI)	Optimal Cut-Off	Sensitivity (95% CI)	Specificity (95% CI)
DNI	50	0.80 (0.66–0.90)	≥0.50	0.57 (0.33–0.76)	0.90 (0.54–1.00)
CRP (mg/L)	50	0.66 (0.50–0.82)	≥124.31	0.57 (0.33–0.76)	0.83 (0.52–0.96)
ESR (mm/h)	50	0.47 (0.31–0.65)	≥28	0.71 (0.50–0.88)	0.38 (0.14–1.00)
WBC (×10^9^/L)	50	0.64 (0.48–0.79)	≥9.97	0.86 (0.67–0.95)	0.48 (0.30–0.87)
PCT (ng/mL)	43	0.50 (0.29–0.70)	≥0.27	0.29 (0.11–0.53)	0.86 (0.08–1.00)

**Table 4:** Comparison of sensitivity/specificity using a fixed cut-off based on culture and clinical outcomes.

Biomarker	n	Sensitivity (Culture/Clinic)	Specificity (Culture/Clinic)
DNI (≥0.6)	85	0.943/0.732	0.84/1.00
CRP (≥5)	85	0.971/0.982	0.06/0.103
ESR (≥14)	85	0.943/0.893	0.16/0.138
WBC (>11)	85	0.657/0.607	0.58/0.655
PCT (≥0.5)	67	0.469/0.370	0.914/0.952

**Table 5:** ROC Summary (AUC for culture and clinical reference standards) and optimal cut-off derived using the culture reference standard (Youden index).

Biomarker	n	AUC (Culture/Clinic)	Youden Cut-Off (Culture)	Sensitivity (Culture)	Specificity (Culture)
DNI	85	0.914/0.921	0.6	0.943	0.84
CRP	85	0.687/0.717	138.67	0.629	0.74
ESR	85	0.643/0.579	48.0	0.6	0.68
WBC	85	0.648/0.677	10.35	0.829	0.48
PCT	67	0.697/0.637	0.39	0.531	0.886

**Table 6:** Pairwise DeLong comparisons of biomarker AUCs (Culture/Clinic).

	DNI	CRP	ESR	WBC	PCT
DNI	—	*p* = 0.0003/*p* = 0.0003	*p* = 0.0000/*p* = 0.0000	*p* = 0.0000/*p* = 0.0001	*p* = 0.0012/*p* = 0.0000
CRP	Δ = −0.227/Δ = −0.204 ^1^	—	*p* = 0.5199/*p* = 0.0545	*p* = 0.5860/*p* = 0.6019	*p* = 0.7362/*p* = 0.0899
ESR	Δ = −0.271/Δ = −0.342	Δ = −0.044/Δ = −0.138	—	*p* = 0.9576/*p* = 0.2539	*p* = 0.1886/*p* = 0.5772
WBC	Δ = −0.266/Δ = −0.244	Δ = −0.039/Δ = −0.040	Δ = 0.005/Δ = 0.098	—	*p* = 0.7140/*p* = 0.6530
PCT	Δ = −0.217/Δ = −0.284	Δ = 0.010/Δ = −0.080	Δ = 0.054/Δ = 0.058	Δ = 0.049/Δ = −0.040	—

^1^ Upper triangle: DeLong *p*-values; lower triangle: ΔAUC = AUC (row)–AUC (column).

## Data Availability

The original contributions presented in the study are included in the article; further inquiries can be directed to the corresponding author.
